# Supplementary material for: Extrachromosomal DNA formation enables tumor immune escape potentially through regulating antigen presentation gene expression
Source: Sci Rep. 2022 Mar 4;12:3590. doi: 10.1038/s41598-022-07530-8 (PMC8897507; doi:10.1038/s41598-022-07530-8)
Supplement: Supplementary file 1 — Supplementary Information. [file 41598_2022_7530_MOESM1_ESM.pdf]

Extrachromosomal DNA formation enables tumor immune escape potentially through regulating antigen presentation gene expression

Tao Wu, Chenxu Wu, Xiangyu Zhao, Guangshuai Wang, Wei Ning, Ziyu Tao, Fuxiang Chen, Xue-Song Liu

Supplementary Table 1: List of datasets and software used in this study

**data/software**	**resource**
TCGA somatic mutation	https://gdc-hub.s3.us-east-1.amazonaws.com/download/GDC-PANCAN.mutect2_snv.tsv.gz
TCGA mRNA expression	https://toil-xena-hub.s3.us-east-1.amazonaws.com/download/tcga_RSEM_gene_tpm.gz
TCGA ecDNA information	Kim H, Nguyen N P, Turner K, et al. Extrachromosomal DNA is associated with oncogene amplification and poor outcome across multiple cancers[J]. Nature genetics, 2020, 52(9): 891-897.
ESTIMATE	R estimate package: https://bioinformatics.mdanderson.org/estimate/rpackage.html
Immune cell infiltration estimation	TIMER webserver: http://timer.comp-genomics.org/, including the results calculated by TIMER, CIBERSORT, quanTIseq, xCell, and MCP-counter algorithms
Leukocyte fraction	Thorsson V, Gibbs D L, Brown S D, et al. The immune landscape of cancer[J]. Immunity, 2018, 48(4): 812-830. e14. (https://gdc.cancer.gov/about-data/publications/panimmune)
Immunophenoscore (IPS)	Charoentong P, Finotello F, Angelova M, et al. Pan-cancer immunogenomic analyses reveal genotype-immunophenotype relationships and predictors of response to checkpoint blockade[J]. Cell reports, 2017, 18(1): 248-262.
Tumor inflammation signature (TIS)	Danaher P, Warren S, Lu R, et al. Pan-cancer adaptive immune resistance as defined by the Tumor Inflammation Signature (TIS): results from The Cancer Genome Atlas (TCGA)[J]. Journal for immunotherapy of cancer, 2018, 6(1): 1-17. Use GSVA to calculate TIS.
Cytolytic activity score (CYT)	Geometric mean of expression of GZMA and PRF1 genes
TCGA samples immune subtype	1.Thorsson V, Gibbs D L, Brown S D, et al. The immune landscape of cancer[J]. Immunity, 2018, 48(4): 812-830. e14. (https://gdc.cancer.gov/about-data/publications/panimmune) 2.Bagaev A, Kotlov N, Nomie K, et al. Conserved pan-cancer microenvironment subtypes predict response to immunotherapy[J]. Cancer Cell, 2021, 39(6): 845-865. e7.
maf2vcf	https://github.com/mskcc/vcf2maf/blob/main/maf2vcf.pl
neoantigen prediction software: NeoPredPipe	https://github.com/MathOnco/NeoPredPipe

Supplementary Table 2: List of genes in gene sets used for GSVA

pathways	genes for GSVA analysis
GO MHC-I	ABCB9,ACE,AZGP1,B2M,BCAP31,CALR,CANX,CD207,CD36,CHUK,CLEC4A,CYBA,CYBB,ERAP1,ERAP2,FCER1G,FCGR1A,FCGR1B,HFE,HLA-A,HLA-B,HLA-C,HLA-E,HLA-F,HLA-G,HLA-H,IDE,IFI30,IKBKB,IKBKG,ITGAV,ITGB5,LNPEP,MFSD6,MR1,NCF1,NCF2,NCF4,PDIA3,PSMA1,PSMA2,PSMA3,PSMA4,PSMA5,PSMA6,PSMA7,PSMA8,PSMB1,PSMB10,PSMB11,PSMB2,PSMB3,PSMB4,PSMB5,PSMB6,PSMB7,PSMB8,PSMB9,PSMC1,PSMC2,PSMC3,PSMC4,PSMC5,PSMC6,PSMD1,PSMD10,PSMD11,PSMD12,PSMD13,PSMD14,PSMD2,PSMD3,PSMD4,PSMD5,PSMD6,PSMD7,PSMD8,PSMD9,PSME1,PSME2,PSME3,PSME4,PSMF1,SAR1B,SEC13,SEC22B,SEC23A,SEC24A,SEC24B,SEC24C,SEC24D,SEC31A,SNAP23,TAP1,TAP2,TAPBP,TAPBPL,VAMP3,VAMP8
GO MHC-II	ACTR10,ACTR1A,ACTR1B,AP1B1,AP1G1,AP1M1,AP1M2,AP1S1,AP1S2,AP1S3,AP2A1,AP2A2,AP2B1,AP2M1,AP2S1,ARF1,CANX,CAPZA1,CAPZA2,CAPZA3,CAPZB,CD74,CENPE,CLTA,CLTC,CTSD,CTSE,CTSF,CTSL,CTSS,CTSV,DCTN1,DCTN2,DCTN3,DCTN4,DCTN5,DCTN6,DNM2,DYNC1H1,DYNC1I1,DYNC1I2,DYNC1LI1,DYNC1LI2,DYNLL1,DYNLL2,FCER1G,FCGR2B,HLA-DMA,HLA-DMB,HLA-DOA,HLA-DOB,HLA-DPA1,HLA-DPB1,HLA-DQA1,HLA-DQA2,HLA-DQB1,HLA-DQB2,HLA-DRA,HLA-DRB1,HLA-DRB3,HLA-DRB4,HLA-DRB5,IFI30,KIF11,KIF15,KIF18A,KIF22,KIF23,KIF26A,KIF2A,KIF2B,KIF2C,KIF3A,KIF3B,KIF3C,KIF4A,KIF4B,KIF5A,KIFAP3,KLC1,KLC2,LAG3,LGMN,MARCHF1,MARCHF8,OSBPL1A,PIKFYVE,PYCARD,RAB7A,RACGAP1,RILP,SAR1B,SEC13,SEC23A,SEC24A,SEC24B,SEC24C,SEC24D,SEC31A,SH3GL2,SPTBN2,THBS1,TRAF6,TREM2
REATOME MHC-I	ANAPC1,ANAPC10,ANAPC11,ANAPC13,ANAPC2,ANAPC4,ANAPC5,ANAPC7,AREL1,ARIH2,ASB1,ASB10,ASB11,ASB12,ASB13,ASB14,ASB15,ASB16,ASB17,ASB18,ASB2,ASB3,ASB4,ASB5,ASB6,ASB7,ASB8,ASB9,ATG7,B2M,BCAP31,BLMH,BTBD1,BTBD6,BTK,BTRC,CALR,CANX,CBLB,CBLL2,CCNF,CD14,CD207,CD36,CDC16,CDC20,CDC23,CDC26,CDC27,CDC34,CHUK,CTSL,CTSS,CTSV,CUL1,CUL2,CUL3,CUL5,CUL7,CYBA,CYBB,DCAF1,DET1,DTX3L,DZIP3,ELOB,ELOC,ERAP1,ERAP2,FBXL12,FBXL13,FBXL14,FBXL15,FBXL16,FBXL18,FBXL19,FBXL20,FBXL22,FBXL3,FBXL4,FBXL5,FBXL7,FBXL8,FBXO10,FBXO11,FBXO15,FBXO17,FBXO2,FBXO21,FBXO22,FBXO27,FBXO30,FBXO31,FBXO32,FBXO4,FBXO40,FBXO41,FBXO44,FBXO6,FBXO7,FBXO9,FBXW10,FBXW11,FBXW12,FBXW2,FBXW4,FBXW5,FBXW7,FBXW8,FBXW9,FCGR1A,FCGR1B,FGA,FGB,FGG,FZR1,GAN,GLMN,HACE1,HECTD1,HECTD2,HECTD3,HECW2,HERC1,HERC2,HERC3,HERC4,HERC5,HERC6,HLA-A,HLA-B,HLA-C,HLA-E,HLA-F,HLA-G,HMGB1,HSPA5,HUWE1,IKBKB,IKBKG,ITCH,ITGAV,ITGB5,KBTBD13,KBTBD6,KBTBD7,KBTBD8,KCTD6,KCTD7,KEAP1,KLHL11,KLHL13,KLHL2,KLHL20,KLHL21,KLHL22,KLHL25,KLHL3,KLHL41,KLHL42,KLHL5,KLHL9,LMO7,LNPEP,LNX1,LONRF1,LRR1,LRRC41,LRSAM1,LTN1,LY96,MEX3C,MGRN1,MIB2,MKRN1,MRC1,MRC2,MYD88,MYLIP,NCF1,NCF2,NCF4,NEDD4,NEDD4L,NPEPPS,PDIA3,PJA1,PJA2,PRKN,PSMA1,PSMA2,PSMA3,PSMA4,PSMA5,PSMA6,PSMA7,PSMA8,PSMB1,PSMB10,PSMB11,PSMB2,PSMB3,PSMB4,PSMB5,PSMB6,PSMB7,PSMB8,PSMB9,PSMC1,PSMC2,PSMC3,PSMC4,PSMC5,PSMC6,PSMD1,PSMD10,PSMD11,PSMD12,PSMD13,PSMD14,PSMD2,PSMD3,PSMD4,PSMD5,PSMD6,PSMD7,PSMD8,PSMD9,PSME1,PSME2,PSME3,PSME4,PSMF1,RBBP6,RBCK1,RBX1,RCHY1,RLIM,RNF111,RNF114,RNF115,RNF123,RNF126,RNF130,RNF138,RNF14,RNF144B,RNF182,RNF19A,RNF19B,RNF213,RNF217,RNF220,RNF25,RNF34,RNF4,RNF41,RNF6,RNF7,RPS27A,S100A1,S100A8,S100A9,SAR1B,SEC13,SEC22B,SEC23A,SEC24A,SEC24B,SEC24C,SEC24D,SEC31A,SEC61A1,SEC61A2,SEC61B,SEC61G,SEM1,SH3RF1,SIAH1,SIAH2,SKP1,SKP2,SMURF1,SMURF2,SNAP23,SOCS1,SOCS3,SPSB1,SPSB2,SPSB4,STUB1,STX4,TAP1,TAP2,TAPBP,THOP1,TIRAP,TLR1,TLR2,TLR4,TLR6,TPP2,TRAF7,TRAIP,TRIM11,TRIM21,TRIM32,TRIM36,TRIM37,TRIM39,TRIM4,TRIM41,TRIM50,TRIM63,TRIM69,TRIM71,TRIM9,TRIP12,UBA1,UBA3,UBA5,UBA52,UBA6,UBA7,UBAC1,UBB,UBC,UBE2A,UBE2B,UBE2C,UBE2D1,UBE2D2,UBE2D3,UBE2D4,UBE2E1,UBE2E2,UBE2E3,UBE2F,UBE2G1,UBE2G2,UBE2H,UBE2J1,UBE2J2,UBE2K,UBE2L3,UBE2L6,UBE2M,UBE2N,UBE2O,UBE2Q1,UBE2Q2,UBE2R2,UBE2S,UBE2U,UBE2V1,UBE2V2,UBE2W,UBE2Z,UBE3A,UBE3B,UBE3C,UBE3D,UBE4A,UBOX5,UBR1,UBR2,UBR4,UFL1,UNKL,VAMP3,VAMP8,VHL,WSB1,WWP1,ZBTB16,ZNRF1,ZNRF2
REATOME MHC-II	ACTR10,ACTR1A,ACTR1B,AP1B1,AP1G1,AP1M1,AP1M2,AP1S1,AP1S2,AP1S3,AP2A1,AP2A2,AP2B1,AP2M1,AP2S1,ARF1,CANX,CAPZA1,CAPZA2,CAPZA3,CAPZB,CD74,CENPE,CLTA,CLTC,CTSA,CTSB,CTSC,CTSD,CTSE,CTSF,CTSH,CTSK,CTSL,CTSO,CTSS,CTSV,DCTN1,DCTN2,DCTN3,DCTN4,DCTN5,DCTN6,DNM1,DNM2,DNM3,DYNC1H1,DYNC1I1,DYNC1I2,DYNC1LI1,DYNC1LI2,DYNLL1,DYNLL2,HLA-DMA,HLA-DMB,HLA-DOA,HLA-DOB,HLA-DPA1,HLA-DPB1,HLA-DQA1,HLA-DQA2,HLA-DQB1,HLA-DQB2,HLA-DRA,HLA-DRB1,HLA-DRB3,HLA-DRB4,HLA-DRB5,IFI30,KIF11,KIF15,KIF18A,KIF20A,KIF22,KIF23,KIF26A,KIF2A,KIF2B,KIF2C,KIF3A,KIF3B,KIF3C,KIF4A,KIF4B,KIF5A,KIF5B,KIFAP3,KLC1,KLC2,KLC3,KLC4,LAG3,LGMN,OSBPL1A,RAB7A,RACGAP1,RILP,SAR1B,SEC13,SEC23A,SEC24A,SEC24B,SEC24C,SEC24D,SEC31A,SH3GL2,SPTBN2,TUBA1A,TUBA1B,TUBA1C,TUBA3C,TUBA3D,TUBA3E,TUBA4A,TUBA4B,TUBA8,TUBAL3,TUBB1,TUBB2A,TUBB2B,TUBB3,TUBB4A,TUBB4B,TUBB6,TUBB8,TUBB8B

Supplementary Table 3: Number of sample information

	cancer type	ecDNA positive	ecDNA negative
Number of TCGA samples with WGS data available for ecDNA detection	BLCA	33	79
	BRCA	29	79
	CESC	15	51
	COAD	4	52
	DLBC	1	6
	ESCA	29	31
	GBM	28	19
	HNSC	39	114
	KICH	1	49
	KIRC	1	43
	KIRP	2	34
	LAML	0	40
	LGG	11	74
	LIHC	4	48
	LUAD	22	121
	LUSC	14	36
	OV	11	34
	PRAD	2	119
	READ	0	18
	SARC	17	19
	SKCM	23	113
	STAD	32	95
	THCA	0	136
	UCEC	20	123
	UVM	1	49
Number of TCGA samples with gene expression data available	BLCA	33	79
	BRCA	29	79
	CESC	15	50
	COAD	4	29
	DLBC	1	6
	ESCA	28	30
	GBM	18	11

	cancer type	ecDNA positive	ecDNA negative
	HNSC	39	113
	KICH	1	49
	KIRC	1	43
	KIRP	2	34
	LAML	0	31
	LGG	11	72
	LIHC	4	46
	LUAD	22	119
	LUSC	14	36
	OV	10	25
	PRAD	2	119
	READ	0	2
	SARC	17	19
	SKCM	23	113
	STAD	27	87
	THCA	0	133
	UCEC	1	8
	UVM	1	48

a

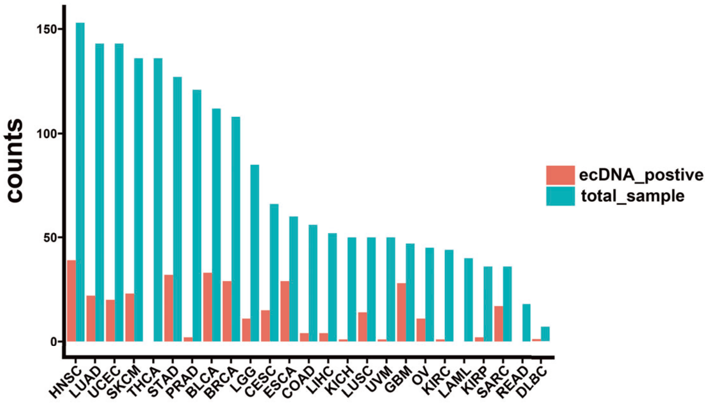

b

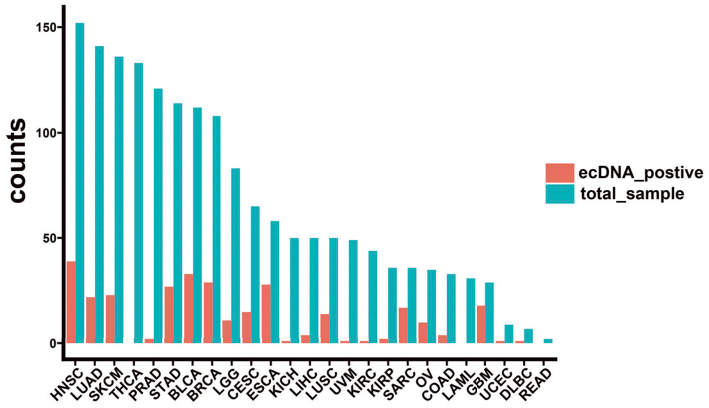

Supplementary Fig. S1 Sample counts distribution of the cancer types with WGS and gene expression data available for ecDNA analysis. a. Total number of cancer samples with ecDNA status information. **b.** Number of cancer samples with both ecDNA status and gene expression information. TCGA cancer type acronyms: THYM (thymoma), ESCA (esophageal carcinoma), BRCA (breast invasive carcinoma), LUAD (lung adenocarcinoma), LUSC (lung squamous cell carcinoma), KICH (kidney chromophobe), STAD (stomach adenocarcinoma), CHOL (cholangio-carcinoma), LIHC (liver hepatocellular carcinoma), PRAD (prostate adenocarcinoma), HNSC (head and neck squamous cell carcinoma), KIRP (kidney renal papillary cell carcinoma), SARC (sarcoma), UCEC (uterine corpus endometrial carcinoma), BLCA (bladder urothelial carcinoma), PAAD (pancreatic adenocarcinoma), CESC (cervical squamous cell carcinoma and endocervical adenocarcinoma), GBM (glioblastoma multiforme), KIRC (kidney renal clear cell carcinoma), SKCM (skin cutaneous melanoma), PCPG (pheochromocytoma and paraganglioma), THCA (thy-roid carcinoma), LGG (Brain Lower Grade Glioma), UVM (Uveal Melanoma), OV (Ovarian serous cystadenocarcinoma), COAD(Colon adenocarcinoma), LAML (Acute Myeloid Leukemia), DLBC (Lymphoid Neoplasm Diffuse Large B-cell Lymphoma), READ (Rectum adenocarcinoma).

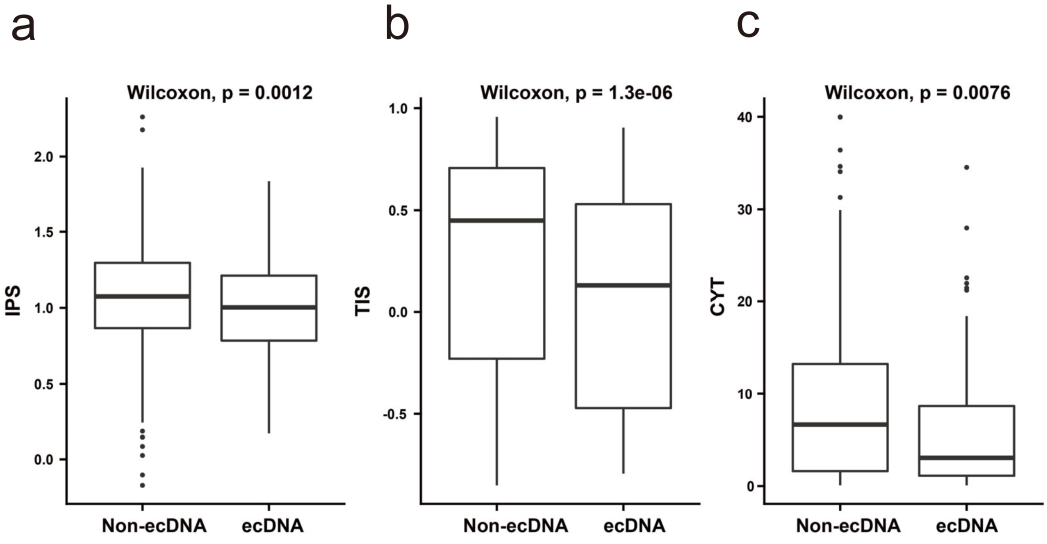

Supplementary Fig. S2 Comparisons of immune infiltration scores calculated by different methods between ecDNA and non-ecDNA samples. a. Immunophenoscore (IPS) was used to measure the immune state of the samples, and was calculated according to Charoentong et al 2017 study. **b.** Tumor inflammation signature (TIS) score was calculat-ed by GSVA using the gene signature described in Danaher et al 2018 study. **c**. Cytolytic activity score (CYT) was calculated as the geometric mean of expression of GZMA and PRF1 genes. Wilcoxon test *P* values are shown.

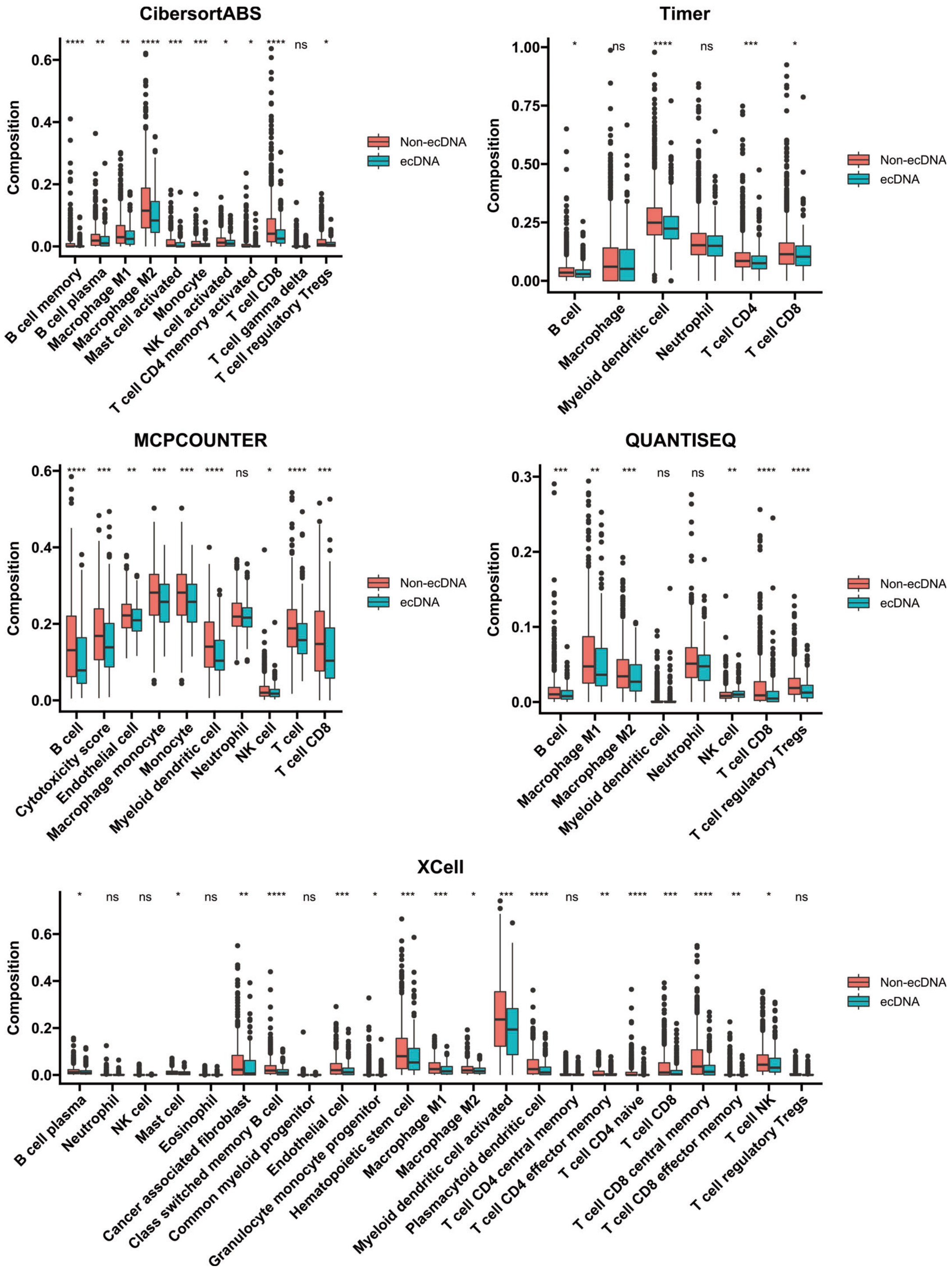

Supplementary Fig. S3 Comparisons of immune cell compositions quantified by five different methods between ecDNA and non-ecDNA samples. Wilcoxon test *P* values are shown. ns: *P*>0.05, *: *P*≤0.05, **: *P*≤0.01, ***: *P*≤0.001, ****: *P*≤0.0001.

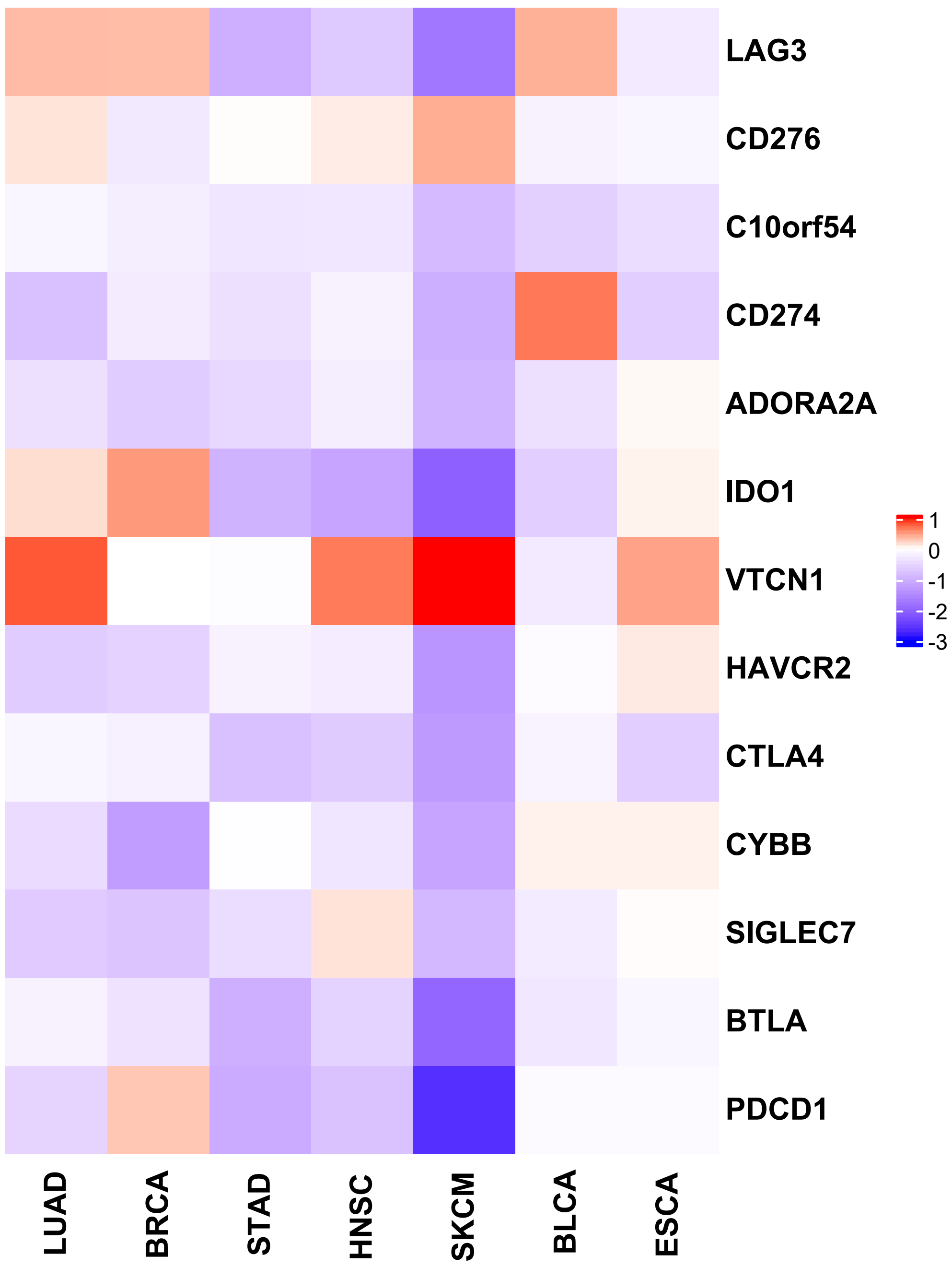

Supplementary Fig. S4 Pan-cancer comparisons of the expression of inhibitory immune checkpoint genes between tumors with and without ecDNA. Heatmap color indicates the difference between the median expression for specific gene in specific cancer type between ecDNA and non-ecDNA samples. Median mRNA expression (in TPM format) of specific gene (row) in samples with ecDNA minus median expression of this gene in samples without ecDNA in a cancer type (column) is reported. TCGA cancer type acronyms: ESCA (esophageal carcinoma), BRCA (breast invasive carcinoma), LUAD (lung adenocarcinoma), STAD (stomach adenocarcinoma), HNSC (head and neck squamous cell carcinoma), BLCA (bladder urothelial carcinoma), SKCM (skin cutaneous melanoma).

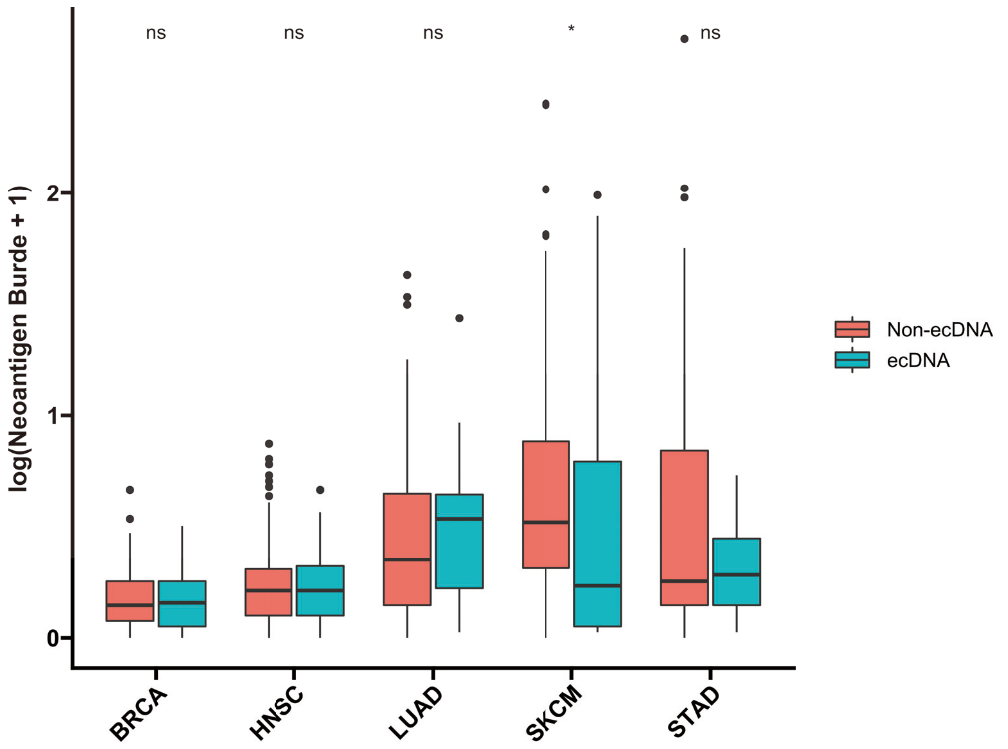

Supplementary Fig. S5 Pan-cancer comparisons of tumor neoantigen burden in tumors with and without ecDNA. Wilcoxon test *P* values are shown. ns: P>0.05, *: $P \leq$ 0.05. TCGA cancer type acronyms: BRCA (breast invasive carcinoma), LUAD (lung adeno-carcinoma), STAD (stomach adenocarcinoma), HNSC (head and neck squamous cell carcinoma), SKCM (skin cutaneous melanoma).

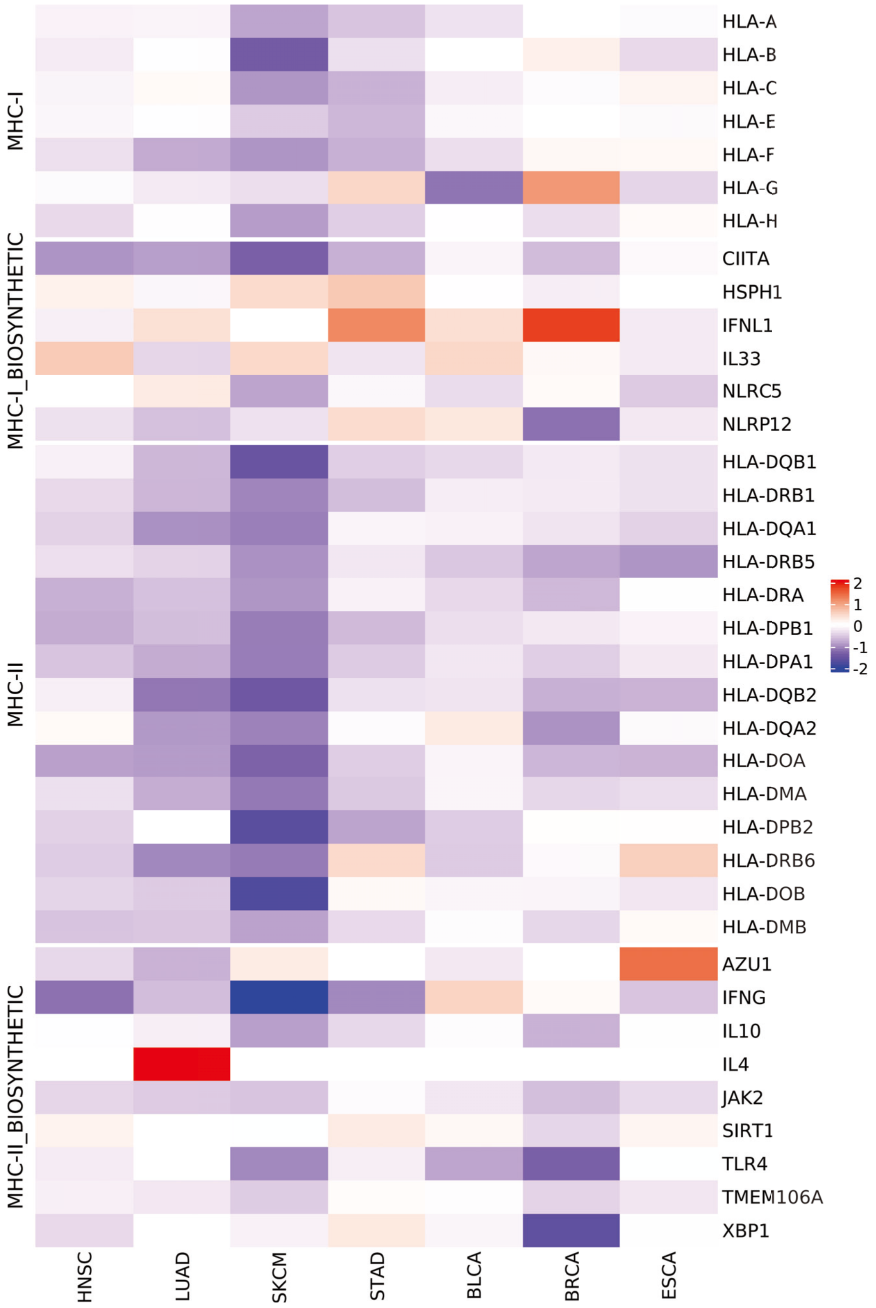

Supplementary Fig. S6 Pan-cancer comparisons of the expression of MHC class I and class II antigen presentation genes in tumor with and without ecDNA. Heatmap color indicates the difference between the median expression for specific gene in specific cancer type between ecDNA and non-ecDNA samples. Median mRNA expression (in TPM format) of specific gene (row) in samples with ecDNA minus median expression of this gene in samples without ecDNA in a cancer type (column) is reported. TCGA cancer type acronyms: ESCA (esophageal carcinoma), BRCA (breast invasive carcinoma), LUAD (lung adenocarcinoma), STAD (stomach adenocarcinoma), HNSC (head and neck squamous cell carcinoma), BLCA (bladder urothelial carcinoma), SKCM (skin cutaneous melanoma).

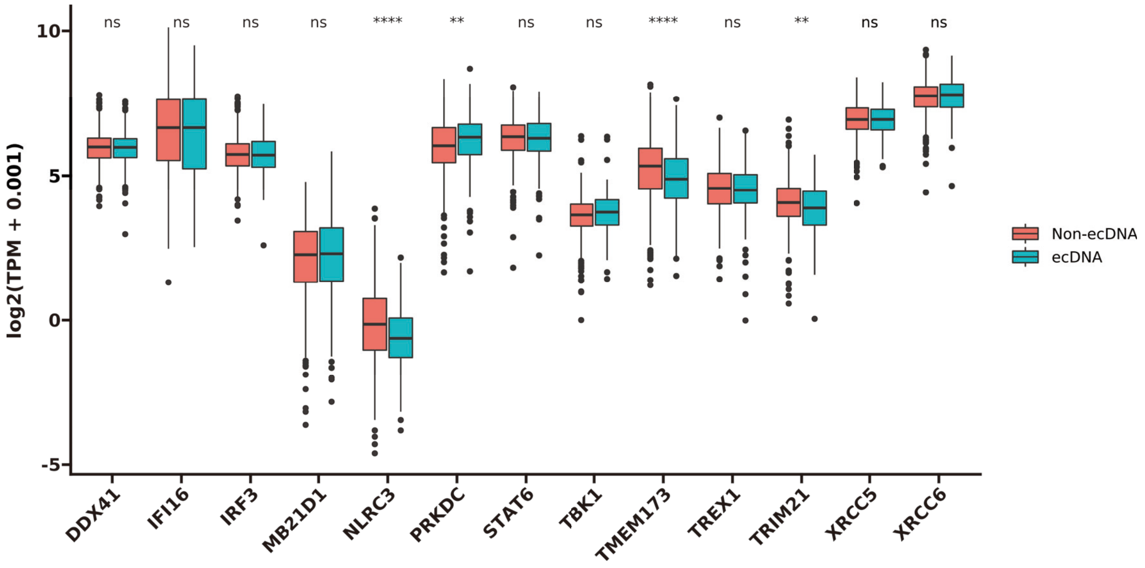

Supplementary Fig. S7 Comparisons of the expression of cGAS-STING pathway genes between tumors with and without ecDNA. Wilcoxon test *P* values are shown. ns: *P*>0.05, *: *P*≤0.05, **: *P*≤0.01, ***: *P*≤0.001, ****: *P*≤0.0001.
